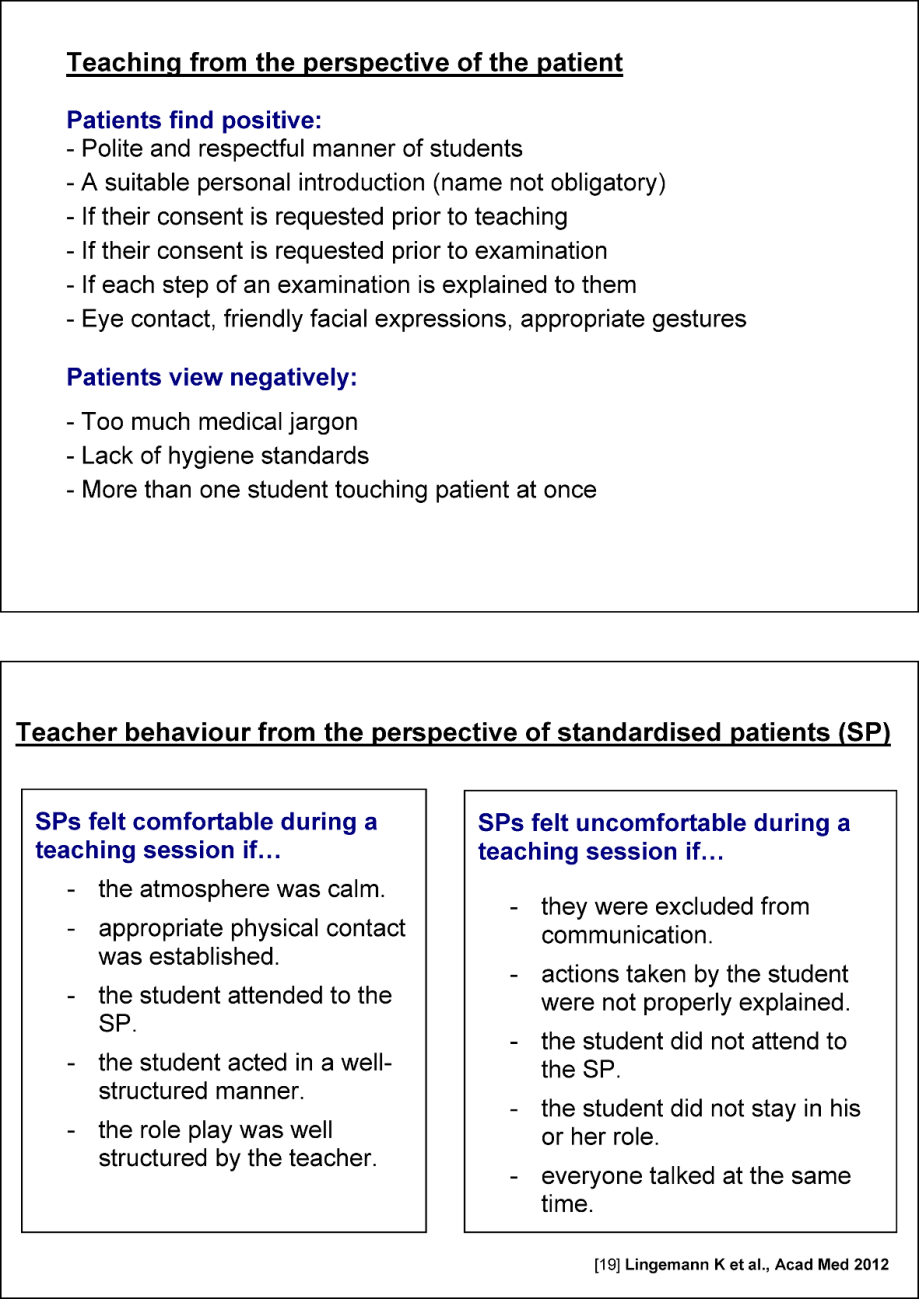# 10 years of didactic training for novices in medical education at Charité

**DOI:** 10.3205/zma001116

**Published:** 2017-10-16

**Authors:** Ulrike Sonntag, Harm Peters, Kai P. Schnabel, Jan Breckwoldt

**Affiliations:** 1 Charité – Universitätsmedizin Berlin, Dieter Scheffner Fachzentrum für medizinische Hochschullehre und evidenzbasierte Ausbildungsforschung, Berlin, Germany; 2 Universität Bern, Institut für Medizinische Lehre, Abteilung für Unterricht und Medien, Bern, Switzerland; 3 Universität Zürich, Medizinische Fakultät, Dekanat, Zürich, Switzerland

**Keywords:** University teacher training, didactics faculty, development qualification

## Abstract

**Introduction: **Many medical faculties are introducing faculty development programmes to train their teaching staff with the aim of improving student learning performance. Frequently changing parameters within faculties pose a challenge for the sustainable establishment of such programmes. In this paper, we aim to describe facilitating and hindering parameters using the example of the basic teacher training (BTT) course at the Charité – Universtitätsmedizin Berlin (Charité).

**Project description: **After sporadic pilot attempts for university education training, basic teacher training was finally established at the Charité in 2006 for all new teaching staff. An interdisciplinary taskforce at the office for student affairs designed the programme according to the Kern cycle of curriculum development, while the Charité advanced training academy provided the necessary resources. Within ten years more than 900 faculty members have completed the BTT (9% of current active teaching staff at the Charité). The BTT programme underwent several phases (piloting, evaluation, review, personnel and financial boosting), all of which were marked by changes in the staff and organizational framework.

Evaluations by participants were very positive, sustainable effects on teaching could be proven to a limited extent.

**Discussion:** Success factors for the establishment of the programme were the institutional framework set by the faculty directors, the commitment of those involved, the support of research grants and the thoroughly positive evaluation by participants.

More challenging were frequent changes in parameters and the allocation of incentive resources for other, format-specific training courses (e.g. PBL) as part of the introduction of the new modular curriculum of the Charité.

**Conclusion:** The sustainment of the programme was enabled through strategic institutional steps taken by the faculty heads. Thanks to the commitment and input by those at a working level as well as management level, the basic teacher training course is today an established part of the faculty development programme at the Charité.

## Zusammenfassung

**Einleitung: **An vielen medizinischen Fakultäten werden Programme zur didaktischen Qualifizierung ihrer Dozierenden aufgebaut, mit dem Ziel, den studentischen Lernerfolg zu steigern. Herausfordernd für die nachhaltige Etablierung derartiger Programme sind oft wechselnde Rahmenbedingungen an den Fakultäten. In dieser Arbeit werden daher exemplarisch förderliche und hinderliche Bedingungen am konkreten Beispiel des Basistrainings Lehre (BTL) an der Charité – Universitätsmedizin Berlin (Charité) dargestellt.

**Projektbeschreibung: **Ausgehend von punktuellen Ansätzen zur hochschuldidaktischen Qualifizierung wurde 2006 an der Charité das Basistraining Lehre für alle neu in der Lehre tätigen Mitarbeitenden etabliert. Eine interdisziplinäre Arbeitsgruppe des Prodekanats für Studium und Lehre entwickelte das inhaltliche Konzept entsprechend des Kern-Zyklus für Curriculumsentwicklung. Die Fortbildungsakademie der Charité stellte die organisatorischen Ressourcen zur Verfügung. Innerhalb von 10 Jahren absolvierten über 900 Mitarbeitende das BTL (9% der aktuell aktiven Dozierenden der Charité). Das BTL durchlief verschiedene Phasen (Pilotierung, Evaluation, Weiterentwicklung, personelle und finanzielle Versteigung), die durch wechselnde personelle und organisatorische Konstellationen gekennzeichnet waren. Evaluationen durch die Teilnehmenden waren sehr positiv, Nachhaltigkeitseffekte auf den Unterricht konnten eingeschränkt nachgewiesen werden.

**Diskussion:** Erfolgsfaktoren für die Verstetigung des Programms waren die institutionelle Rahmensetzung durch die Fakultätsleitung, das Engagement der beteiligten Akteure, die Unterstützung durch Drittmittel und die durchweg positive Evaluation durch die Teilnehmenden. Hinderlich erwiesen sich die sich wandelnden Rahmenbedingungen sowie die Allokation der Ressourcen für formatspezifische didaktische Qualifikation im Rahmen der Einführung des Modellstudiengangs Medizin.

**Fazit: **Die Verstetigung des Programms wurde durch institutionelle Weichenstellungen der Fakultätsleitung ermöglicht. Durch den hohen Einsatz und das Engagement der auf Arbeits- wie auf Leitungsebene beteiligten Akteure ist das Basistraining Lehre heute etablierter Bestandteil des hochschuldidaktischen Qualifizierungsprogramms der Charité.

## Authors

The authors K. Schnabel und J. Breckwoldt share the authorship. 

## 1. Introduction

Teaching quality has a significant impact on the learning success of students [1]. For this reason, many medical faculties have set up didactic programmes for qualifying teachers [2], [3], [4]. The sustainable, broad implementation of such programmes poses a huge challenge for faculties, especially when the programme is intended for a large group of novice teaching staff. Knowledge of supportive as well as hindering factors is therefore beneficial. This project report intends to provide an overview of helpful and challenging factors during the introduction of basic teacher training (BTT) at the Charité – Universitätsmedizin Berlin (Charité). It describes the conception, piloting, evaluation and continued development of the training course. The project was conceived along the six steps of curriculum development by Kern [5]; this report is therefore also structured in this way. Following on from this, positive and negative factors for the sustainable establishment of the programme are discussed.

## 2. Project description

### 2.1. Starting point

Starting out from a more general didactics programme for post-doctoral staff, an informal “taskforce faculty development” drew up a concept for the BTT in 2005. The taskforce had come together as an initiative of clinical and non-clinical physicians which was then incorporated into and supported by the Dean’s office for educational affairs and the Charité advanced training academy.

Literature research yields little evidence for a specific approach to training for new teachers. In the BTT, basic didactic skills, an understanding of the role of the teacher along with methods for lesson planning and execution are the core objectives. The trainers on the course should be seen as colleagues, “senior teachers” who are passing on their knowledge to “junior teachers”.

#### 2.2. Conception

The design of the curriculum is based on Kern’s 6 step cycle [5].

##### 2.2.1. Problem identification and general needs assessment (Kern Step 1)

In 2004, a 20-hour training course for medical educators was introduced as mandatory for post-graduate teaching staff. It was regarded by participants as useful, however, it was too propaedeutic for their career status and for this reason not helpful at the time of completion. 

From the perspective of the undergraduate students of the Charité at that time teaching staff were rated very positively with regards to their motivation, friendliness and competency (data from teaching evaluations in 2004-2006). Students, however, requested more practically oriented teaching, more knowledge of the curricular structure, clear communication of learning objectives, as well as more punctuality from teachers.

##### 2.2.2. Needs assessment for targeted learners (Kern Step 2)

In 2005 a survey was carried out of the didactic qualifications of research staff in surgical disciplines (n=30). Most respondents reported having a positive attitude towards teaching and felt that they were sufficiently prepared for their teaching. On average, they had completed 16 hours of training. Nevertheless, 90% of respondents stated the need for further training. They wanted more techniques for lesson design and presentation, as well as strategies for conflict mediation. With regard to trainers, an equal mix of medical and didactics experts was favoured.

Due to the necessities of different teaching fields (non-clinical basic science vs. clinical teaching with patients), it made sense to offer a “non-clinical” version (with the focus on presentation, interaction and visualization) and a “clinical” version (including bedside-teaching).

##### 2.2.3. Goals and objectives of training courses (Kern Step 3)

The faculty development taskforce agreed on the following main learning objectives:

The participants of a basic teacher training course should, on completion of the course, be able to:

1. base the planning of their teaching on learning theory
2. give a description of the most common strategies for learning and teaching
3. apply different strategies of learning and teaching to their own teaching
4. provide structured and constructive feedback
5. have acquired a positive-reinforcing attitude towards the learner
6. have acquired an understanding of their own function/ position as a role model

The specific learning objectives of the basic teacher training course are listed in table 1 (Tab. 1).

##### 2.2.4. Educational Strategies (Kern Step 4)

BTT comprises 20 teaching hours over two days (9am to 5pm, each with 9 teaching units) with a week’s gap, plus two further hours preparation for each day of attendance. Assigning full days of attendance to the course ascertained that participants were released completely from clinical or other duties on course days. The ratio of basic didactics and microteaching exercises started out as 75:25 in the pilot phase and was later changed to 50:50, according to the participants` feedback.

Training courses take place during and outside of the semester, so that participants and institutions are able to plan flexibly. It was decided that the group size would be 7-12.

Table 2 (Tab. 2), table 3 (Tab. 3) and table 4 (Tab. 4) show the design of the course regarding topics, their execution and time plan. 

##### 2.2.5. Implementation of the curriculum (Kern Step 6)

Each year, around 300 new staff involved in teaching are employed at the Charité (approx. 20% of these are non-clinicians). On signing their contract, participants are informed of the obligatory basic teacher training course. Teaching coordinators are informed about the course in a central semester preparation meeting with the Dean and additionally via email. The administration of the courses was initially carried out by the Charité training academy, but this is now managed by the faculty development department at the Dieter Scheffner Centre. As of 2013 the course is accredited by the Berlin Medical Board with 20 credit points. 

**Figure 1:**
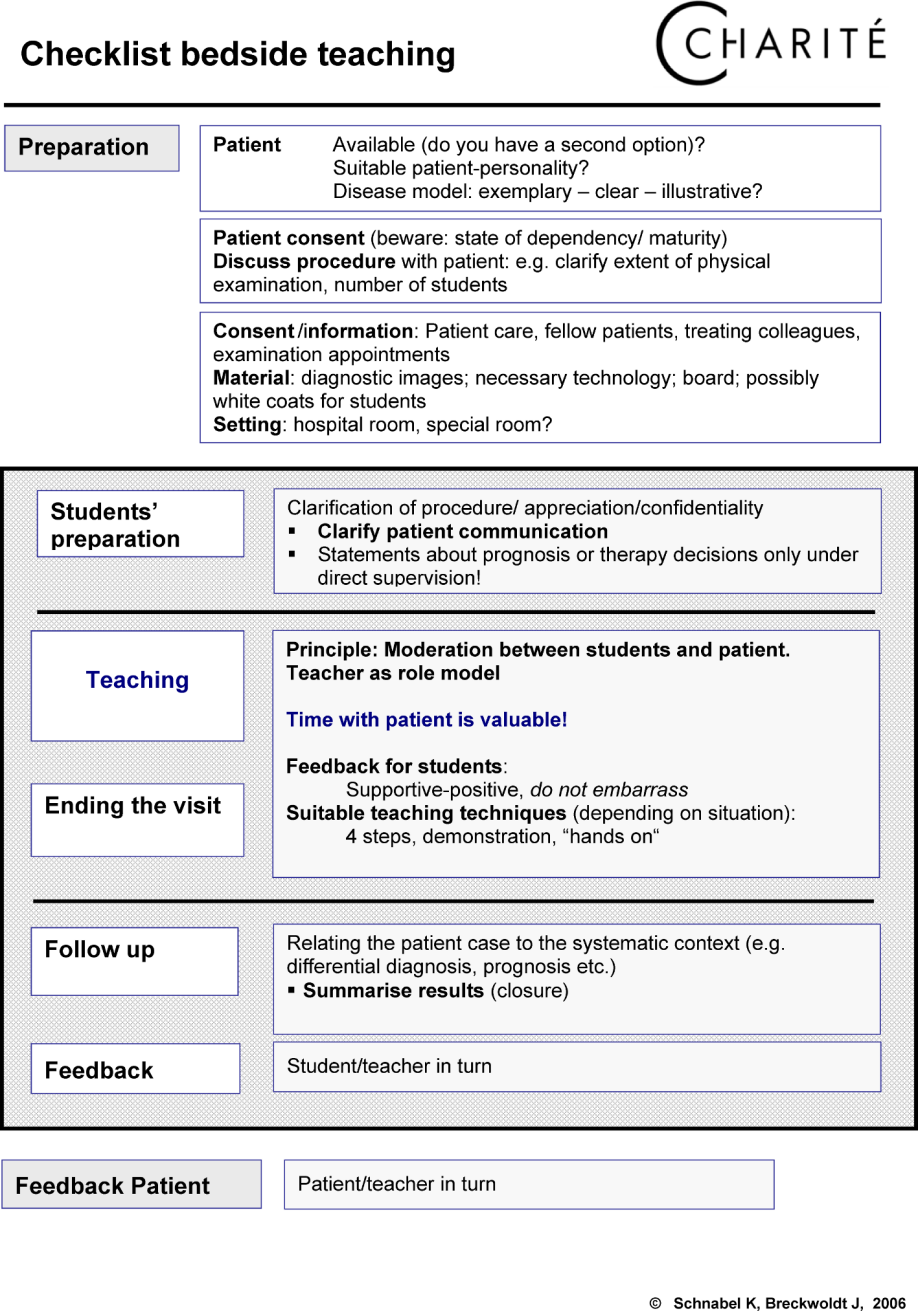


Clinics and institutions allow staff to attend the course during their working hours. During the introductory phase 2006-2010, departments were rewarded for each member of staff who attended the course with €200 “performance merit funding”. This funding was later no longer available. Until 2012 the basic pedagogy section of the course was taught by external trainers. The clinical sections with the focus on bedside-teaching is carried out by experienced clinicians with various educational qualifications. During the introductory phase, trainers each took turns to spend time in the other sections of the course. For sustainable results, participants of the clinical course were given a pocket version of the “Checklist for Bedside Teaching” (see figure 1 (Fig. 1)) and “Teaching from the Patient’s Perspective” (see figure 2 (Fig. 2)).

Since 2012 the courses have been carried out, supported by funding from the Ministry for Education and Research (BMBF) as part of the Quality Pact for Teaching (Reference no. 01PL11036), by a scientific fellow (Educational specialist and psychologist) together with staff from the Dieter Scheffner Centre, who are also clinicians, as part of their routine job. 

Altogether more than 900 members of staff at the Charité have completed the course, which comprises 9% of current teaching staff. Compared to this, 26% of all teaching staff at the Charité are qualified for the “PBL” method and 11% for teaching communication skills and teamwork. Table 5 (Tab. 5) shows the number of basic teacher training courses and attendees per course variant and year.

##### 2.2.6. Evaluation and Feedback (Kern Step 6)

Immediately after the course, participants handed in a written evaluation. The questionnaire has changed several times over the course of the 10 year programme due to personnel changes. Respondents were asked about their subjective satisfaction with the course and the perceived improvement of their teaching skills.

In the pilot phase a semi-open questionnaire was used, as specific comments were felt to be most helpful for curriculum development. For overall quality rating, an interval scale (0-10) was used. In the introductory phase, BTT trainers were also requested to provide a written evaluation of the course.

In order to measure long-term impact, a random selection of participants (n=40) from the year 2007 were surveyed 8 to 15 months after the course on their retrospective view of the training. 

The questionnaire focused on attitudes to teaching as well as teaching techniques used before and after BTT.

As a partial approach to evaluate effects of the BTT, a quasi-randomised, blinded “matched-pair” study was carried out, in which teaching staff of an practical training in emergency medicine were compared, depending on whether or not they had completed BTT. The outcome parameters were student assessment results from three OSCE stations and their course evaluations by questionnaire [6].

By way of example, the evaluation data from the introductory years (2006-2007) and the years 2014-2015 are shown here. In 2006 the overall rating was very positive (return rate 96%, median for the general didactics section: 8/10, for the practical section with microteaching: 9/10). In the open comments respondents rated positively the practical nature of the course, the connection of theory and practice, the appreciation of teaching staff, received feedback and the structure of the course. They wanted more time and space for more extended role play activities. The medical teaching theory section was considered to be too long and a better synchronization of theoretical with practical content was requested. The commitment and positive role modelling of trainers was noted in particular by respondents. In the view of BTT trainers, there was a very positive atmosphere on the course, despite its obligatory nature. Many respondents saw the establishment of the course as an important gesture of acknowledgment by the faculty of the importance of teaching. In particular, clinical practical aspects of the course were seen to have filled a major deficit. The short time available for practical exercises was felt to be problematic. Participants had insufficient time for their own activities and individual feedback. 

In 2014 and 2015, data from 121 participants was collected (90% return rate). Respondents rated the course as part of the evaluation by the Berlin Chamber of Physicians on a scale of 1-6 (1 excellent, 6 completely unsatisfactory) for 12 aspects of the course, e.g. the content and their own knowledge gains. All aspects were rated at 2 (median) or better. The clinical relevance of the course in the clinical version was rated higher.

The retrospective survey from 2007 yielded a 40% return. Respondents reported improved attitudes to teaching and more frequent use of case examples in lessons (93% and 62%). The use of other teaching strategies, however, had hardly changed. The motivation to teach remained high. Respondents reported being less happy with their own teaching after BTT than before.

The comparison of teaching staff in the emergency medicine teaching with or without BTT did not show any improvement after BTT.

Due to the evaluations and to frequent changes in parameters, the BTT continued to be developed. The course outline for 2007 was changed according to the evaluations for the pilot phase. The clinical part (bedside-teaching) was expanded from half a day to a full day, and the didactic elements were aligned more to the clinical part. In the role plays for example, more reference was made to general didactic theory while didactic elements from the clinical part were transferred to the general didactic part.

The topic of “studying and teaching at the Charité” was given more time due to an increase in teaching staff’s needs during the introduction of the modular medical degree. As well as this, since 2016, undergraduate students have been invited to the courses for approx. 30 mins in order to comment on their perspective, in particular with regard to the specific situation with the evolving new modular curriculum at the Charité.

## 3. Discussion

In a systematic approach based on Kern, a basic training course was conceived, piloted, evaluated and further developed with the aim of preparing new scientific staff for their role as teacher. Educational objectives correlate with national recommendations [7], [8], [9].

### 3.1. Facilitating factors

Although Deans and Vice Deans changed several times, the continued commitment of individuals and the training academy at the Charité led to a sustained core concept for the programme. Especially in its first years, this was key to its success. Through the acquisition of grant funding (Ministry for Education and Research) and the consolidation of the programme in the Faculty Development Programme at the Dieter Scheffner Center, it was possible to establish BTT long term. Continuous positive evaluations by participants and word of mouth advertising led to the success of the training. The learning gain from the course is rated as good to very good.

These results correspond to the results of a systematic review of evaluations for teacher training [10], in which respondents stated that they were generally very satisfied with university teacher training courses, and reported gains in their teaching skills and knowledge. A reported lack of satisfaction with their own teaching following the BTT course could be explained as a consequence of increased expectations of one’s own teaching.

Both versions of the course are rated equally. As expected, the aspects of clinical relevance in the clinical version is rated significantly better [11].

Indirect and slightly pronounced indications point to the long-term value of BTT. In our studies, limitations exist with regard to an already existing ceiling effect, the restriction to mainly standardized skills training in emergency medicine as well as the early point of measuring. A pre- post-design for an efficacy review of the training was not possible. It is to be expected that new teaching strategies will need to be integrated into routines before sustained improvement can be observed.

The question of the long-term value of an introductory course for teaching is still open. The subjective gain in competency and knowledge was measured together with the satisfaction with the course. The study on the retrospective rating of the course is not representative. There was a tendency for the attitude to teaching to change after BTT, but this was not the case for the methods used. For an objective perspective, a pre-post-test would be necessary. Dennick showed that participants of a two-day didactics course improved their teaching methods and their attitudes to teaching [12]. Hofer et al [4] were able to show objective improvements in teaching after completion of a five day teacher training course. 

#### 3.2 Hindering factors

Variations in the numbers of participants in BTT correspond to changes in parameters as part of the switch from the traditional to the new modular curriculum at the Charité. According to recommendations by the Science Board on the further development of medical curricula [13], faculty development is a fundamental factor for the successful implementation of modular curricula. The focus of teacher training at the Charité has in the last few years been on the teaching formats “problem-based learning” and “communication, interaction and teamwork”. In the qualification courses for these formats, basic didactic elements are also included. It is possible that departments send staff to qualify for these teaching methods because on completion, staff are allowed to teach PBL and communication straight away and their teaching appointments benefit departments for the allocation of funding. 

Furthermore, the teaching burden for many departments during the years of adjustment to the new curriculum, during which three medical degree courses ran parallel, was enormous, so that departments tended to send their staff less to general didactic courses.

The small percentage of present teaching staff at the faculty who have completed the BTT highlights firstly a high staff turnover in the last few years, but also shows that the obligation for new staff to attend is not being implemented or that non-attendance is being permitted. Our data shows that the attendance for BTT has risen significantly again in the last two years, which can partly be explained by the completed implementation of the modular curriculum.

It is also possible that the moratorium for performance merit funds for course attendance together with the departure of the BTT initiators has had a negative impact on the visibility and appeal of the course. Teaching in medical faculties is increasingly in competition with patient care and the focus on research activities. This could also explain a decrease in the numbers of course participants [14]. Huwendiek et al hold that in particular the lack of academic recognition and institutional/financial support pose a challenge for teaching activities [15].

Steinert et al identify similar barriers for the utilization by clinical teaching staff of faculty development programmes [16], particularly the clinical setting with its high demands and lack of time are seen as constraining factors. 

#### 3.3. Further development of the basic teacher training course

In future, further development of the BTT design will concentrate on the integration of eLearning-elements as part of a blended learning concept. Attendance times can then be used to maximise the application of learned knowledge, and participants will have greater flexibility with regards to self-study.

For didactic qualifications to be effective at a faculty, the training of a critical number of teaching staff is crucial [7]. Appeal factors for the participation of staff and their departments need to be identified and strategically better implemented. In order to accommodate changing demands on staff (“shift from teaching to learning”) [17], [18], a new evaluation survey specific to BTT was developed (see attachment 1 ). Through this, a dynamic and participant-oriented further development of BTT is hoped for.

The inclusion of student input in the BTT concept is seen as an important enhancement. Learners become tangible as the recipients of university teaching, and awareness for the mutual and reciprocal responsibility for learning is strengthened. Building on BTT, a new module “advanced-teaching” was introduced in 2016. This course aims at more experienced teachers who wish to further qualify in the area of teaching, for example as part of their “Habilitation” (post-doctorate) pathway. The first feedback on this second level qualification course was positive.

## 4. Conclusions

With a systematic approach to programme development and implementation, the Charité has succeeded in conveying basic teaching techniques and a positive attitude to teaching to new scientific staff. The establishment of this programme was facilitated by an institutional framework provided by the Dean and the Vice Dean for Educational Affairs along with strong commitment on the part of those involved. Basic teacher training has successfully been established as a starting point for new staff and is a fixed component of the faculty development programme at the Charité.

## Competing interests

The authors declare that they have no competing interests. 

## Supplementary Material

Evaluation form for Basic Teacher Training (since 11/2015)

## Figures and Tables

**Table 1:**
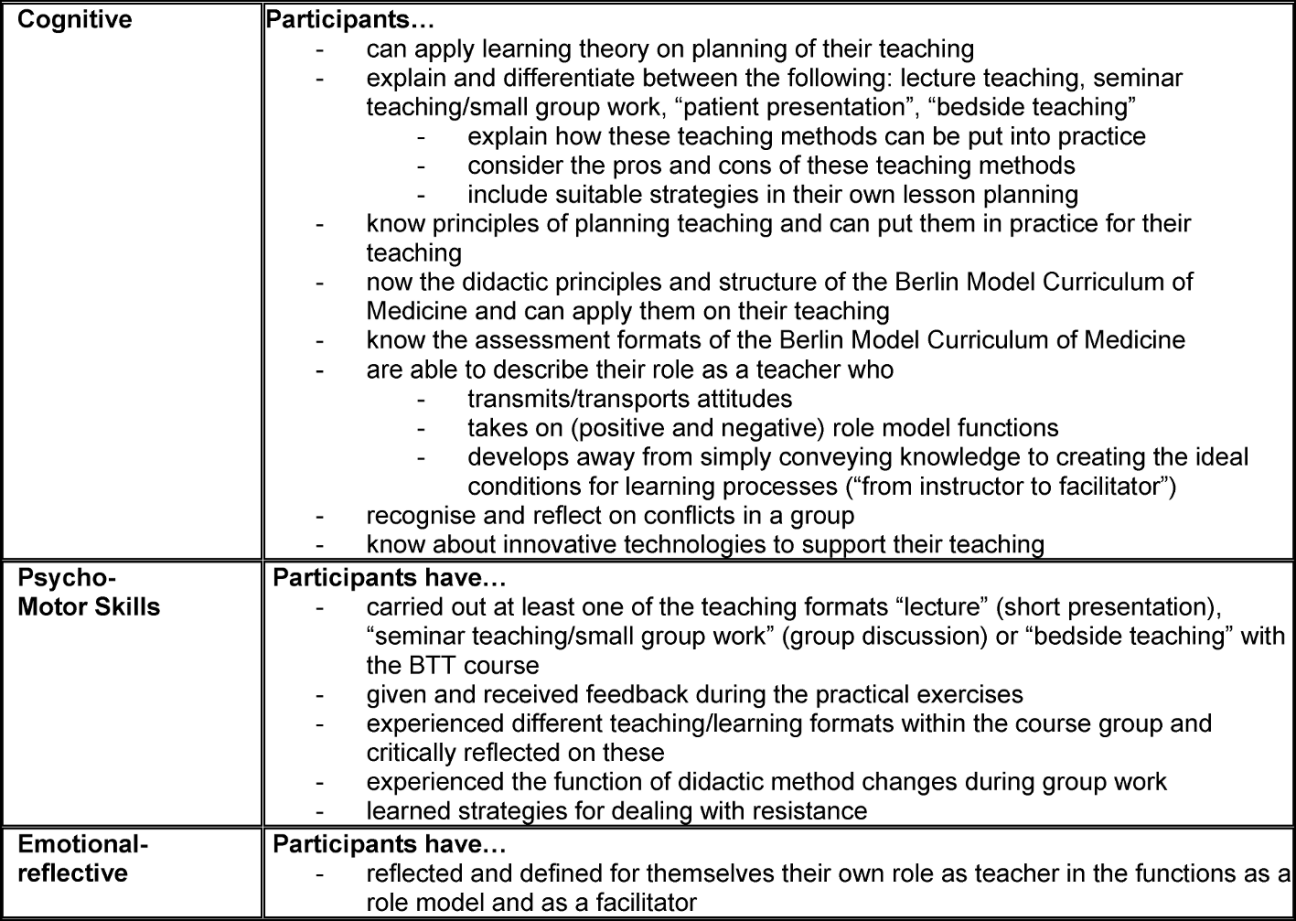
Specific learning objectives Basic Teacher Training (BTT)

**Table 2:**
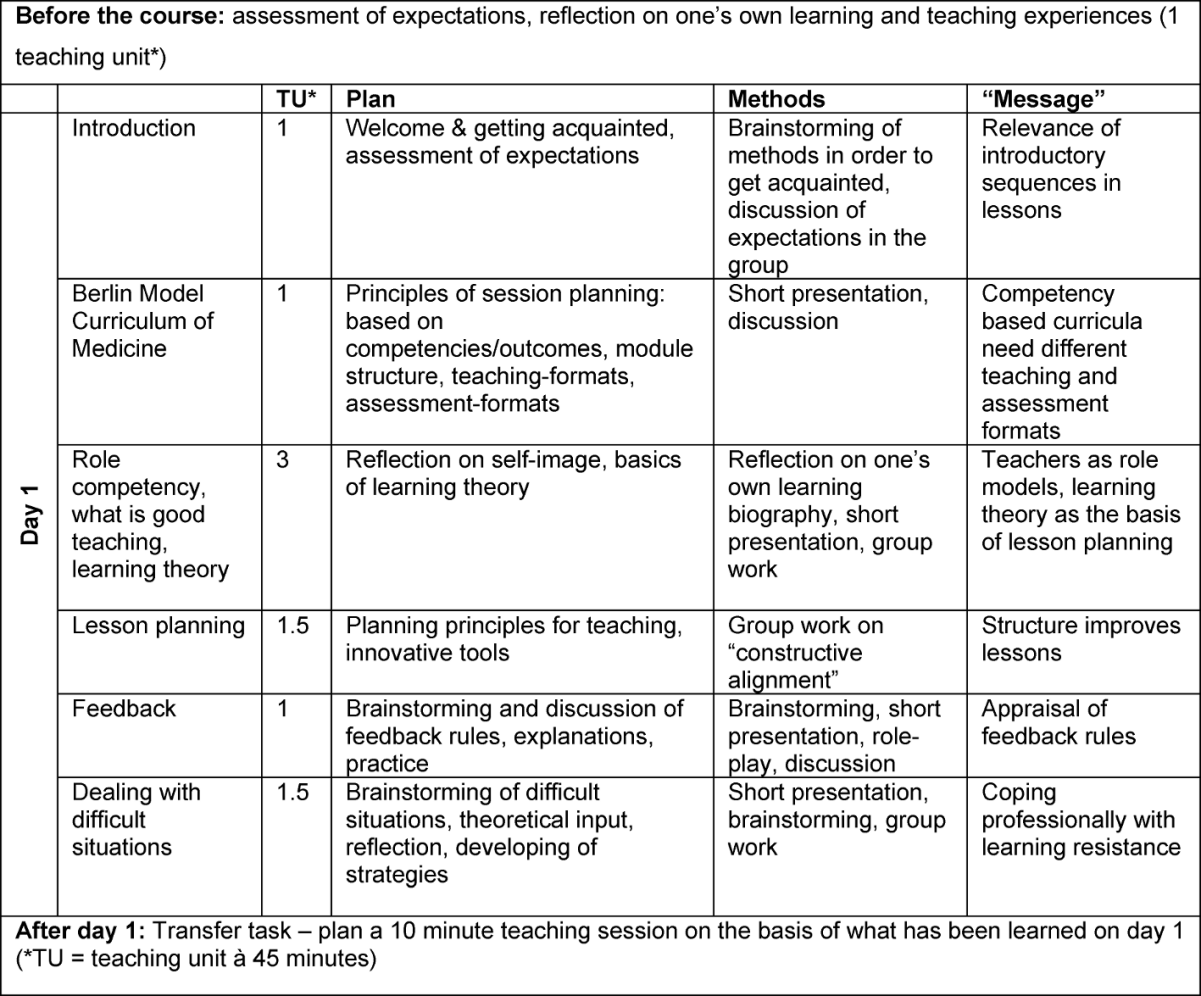
Schedule day 1 of the Basic Teacher Training (BTT), including preparation and follow-up

**Table 3:**
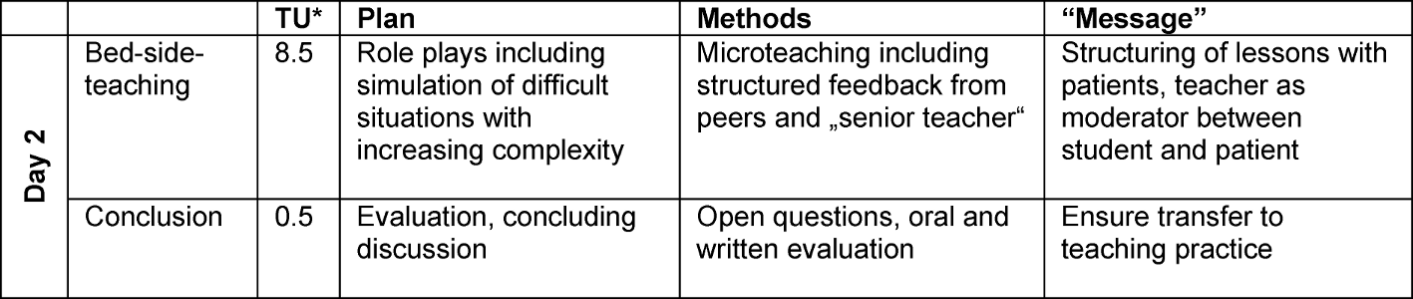
Schedule day 2 clinical version of Basic Teaching Training (BTT)

**Table 4:**
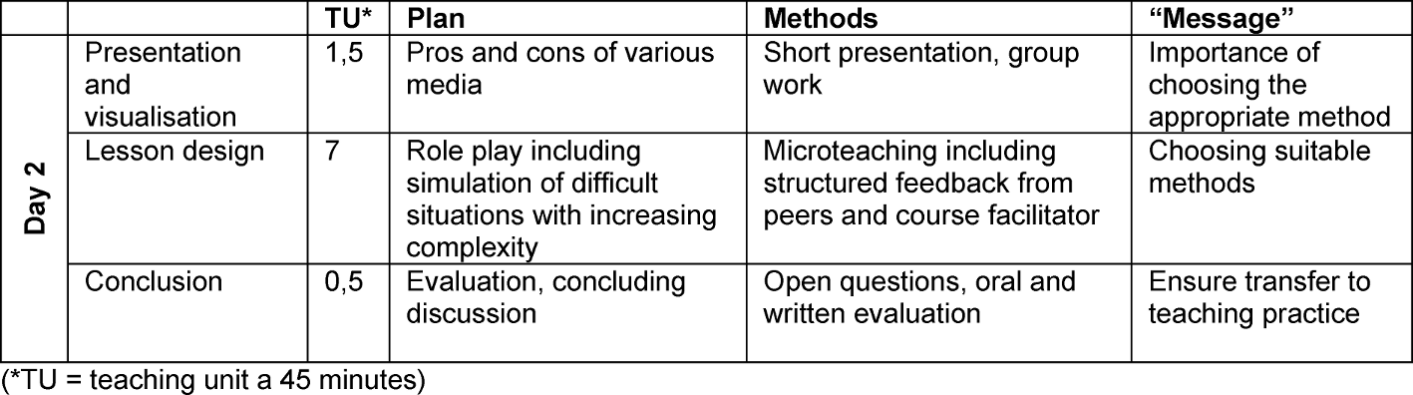
Schedule day 2 non-clinical version of Basic Teacher Training (BTT)

**Table 5:**
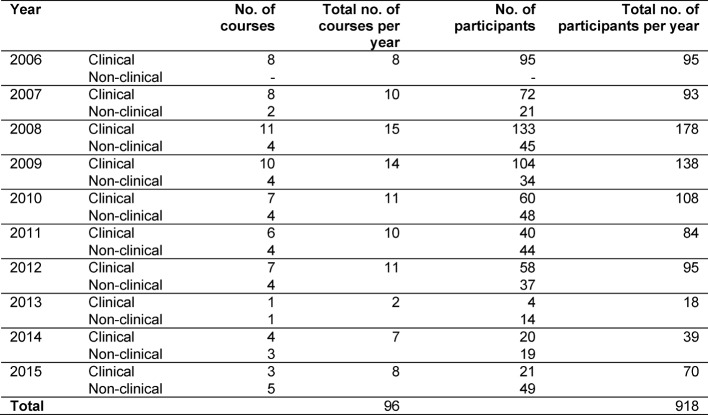
Number of courses and participants per year in relation to course version

**Figure 2:**