# Supplementary material for: 10 years of didactic training for novices in medical education at Charité
Source: GMS J Med Educ. 2017 Oct 16;34(4):Doc39. doi: 10.3205/zma001116 (PMC5654120; doi:10.3205/zma001116)
Supplement: Evaluation form for Basic Teacher Training (since 11/2015) [file JME-34-39-s-001.pdf]

How long have you been teaching?

- ☐ I have not yet taught
- ☐ 1-2 years
- ☐ 3-4 years
- ☐ More than 4 years

In which kind of clinic or institute do you work?

- ☐ Clinical
- ☐ Clinical, theoretical
- ☐ Basic medical science
- ☐ Other: ____________________________

In which teaching formats have you already taught? (more than one answer possible)

- ☐ Small group (e.g. Communication course, PBL, bedside teaching)
- ☐ Middle sized groups (e.g. seminar)
- ☐ Large group (e.g. lecture)

What motivated you to take part in this training course? (more than one answer possible)

- ☐ Own interest
- ☐ Obligated to by teaching coordinator / supervisor
- ☐ Other: ____________________________

Please state to what extent you agree with the following:

	Completely agree	Agree	Partly agree	Don't really agree	Do not agree
I learned new teaching methods which I can apply in my teaching sessions	☐	☐	☐	☐	☐
The structure of the course made sense for me	☐	☐	☐	☐	☐
I found the ratio of theory content to practical exercises appropriate	☐	☐	☐	☐	☐
I feel well prepared for my teaching	☐	☐	☐	☐	☐
I can prepare my teaching so that students benefit	☐	☐	☐	☐	☐
I have a good overview of the medical curriculum at the Charité	☐	☐	☐	☐	☐
The materials and media used in the course helped me understand the topics	☐	☐	☐	☐	☐
I would recommend this course to my colleagues	☐	☐	☐	☐	☐
I am generally very satisfied with the course	☐	☐	☐	☐	☐
I would like to spend more of my working time on teaching (including preparation and follow-up)	☐	☐	☐	☐	☐

	Much too long	Too long	Appropriate	Too short	Much too short
The duration of the course was	☐	☐	☐	☐	☐

What did you especially like about the course?

What would you do differently if you were teaching this course?

Would you like to take part in more faculty development courses? If so, on what topics?

Thank you!
